# Disparities in Health Outcomes and Access to Care Between Sexual Minority and Heterosexual Hispanic Adults: A Non-Monolithic Approach

**DOI:** 10.1007/s10903-025-01684-z

**Published:** 2025-03-26

**Authors:** Gilbert Gonzales, Eric Connelly

**Affiliations:** 1 Department of Medicine, Health & Society Program for Public Policy Studies, Vanderbilt University, Nashville, TN USA; 2 Vanderbilt University, Nashville, TN USA

**Keywords:** Lesbian, Gay, Bisexual, Queer (LGBQ), Hispanic ethnicities, Access to care, Intersectionality

## Abstract

A large body of research has documented disparities in health and access to care experienced by sexual minorities and Hispanic populations in the United States. Very few population-based studies have examined health outcomes at the intersections of sexual orientation and Hispanic ethnicity– and large research gaps remain on the health of lesbian, gay, bisexual, and queer/questioning (LGBQ+) Hispanic communities by specific Hispanic ethnicities and/or ancestries. The objectives of this study are to compare health outcomes and access to care between LGBQ + adults and heterosexual adults by specific Hispanic ethnicities. We use representative data from Hispanic adults identifying as sexual minority (*n* = 768) or heterosexual (*n* = 26,036) in the 2013–2018 National Health Interview Surveys. Descriptive statistics and chi-squared tests were used to compare demographic characteristics (e.g., age, sex, relationship status, survey language, and educational attainment) across ethnicities by sexual minority status. Logistic regression models evaluated differences in self-reported health outcomes and barriers to care. After adjusting for sociodemographic characteristics, compared to their heterosexual peers, sexual minority Mexicans, Mexican Americans, and Central/South Americans were significantly more likely to report moderate to severe psychological distress and unmet mental health care needs due to cost. Sexual minority Cubans were more likely to report having a chronic health condition compared to their heterosexual peers. This study demonstrates the importance of approaching LGBQ + and Hispanic health with non-monolithic perspectives. Future research should continue to leverage community-based research, large-scale quantitative surveys, and qualitative research to help inform targeted interventions that advance LGBQ + Hispanic health equity.

## Background

A large and growing body of population health research has documented health disparities experienced by lesbian, gay, bisexual, queer, and/or questioning (LGBQ+) populations [1, 2]. Prior research has documented that LGBQ + populations are more likely to report adverse health outcomes as a result of minority stress [3], which postulates that structural and interpersonal discrimination can translate to worse mental health outcomes and adverse coping behaviors among LGBQ + populations [4]. Meanwhile, LGBQ + populations also experience financial barriers to care [5] and challenges accessing LGBQ + affirming health care providers [6]. Such barriers to care can exacerbate and widen LGBQ + health disparities which may be costly and preventable.

Meanwhile, Hispanic populations in the United States also experience unique health advantages, health disparities, and barriers to care. For instance, Hispanics have longer life expectancies and lower death rates due to cancer and cardiovascular disease despite their relatively lower socioeconomic status compared to their non-Hispanic White counterparts [7]. Researchers investigating the so-called Hispanic mortality paradox speculate that first-generation immigrant Hispanic populations tend to be younger, healthier, and exhibit better health behaviors prior to assimilation– which may overstate overall better health outcomes [8]. Yet, Hispanics also have a higher prevalence of diabetes and kidney-related diseases compared to their non-Hispanic White peers [7]. Hispanics are also more likely to be uninsured (17.7% in 2021) compared to their non-Hispanic White counterparts (5.7% in 2021) [9]. Among Spanish-speaking Hispanics, language barriers can also facilitate barriers to culturally competent care [10]. 

Continued research on the growing and diverse Hispanic population is still needed in the United States. According to the Pew Research Center [11], the Hispanic population grew from 50.5 million in 2010 (when the US Census Bureau updated questions enabling respondents to identify as a broader and more detailed set of Hispanic sub-ethnicities) to 62.1 million in 2020. This 23% increase was only outpaced by a faster-growing Asian population [11]. The current study’s contribution is that it examines sexual orientation-based disparities within the largest racial/ethnic minority group in the nation: the Hispanic population.

## Conceptual Framework

Very few studies have examined health and access to care at the intersections of LGBQ + status and Hispanic ethnicity. According to prior investigations, LGBQ + Hispanic adults are more likely to be uninsured [12], have worse self-rated health [13], exhibit excessive alcohol consumption [14], and higher levels of sexually transmitted infections [15] — including HIV — than their heterosexual Hispanic peers. Much less research has examined LGBQ + Hispanic health by specific Hispanic ethnicities and ancestries — thus ignoring non-monolithic approaches towards studying LGBQ + Hispanic health. While large, Hispanic populations are very diverse with varying histories and experiences in the United States. For instance, some Cuban Americans are political refugees or descendants of Cubans who fled the communist regime led by Cuban dictator Fidel Castro in the mid-twentieth century. As a result, there are large Cuban American communities in southern Florida [16]. Mexican Americans make up the largest Hispanic subpopulation and reside primarily in the southern and western United States following land annexations and/or immigration into the US [16]. Puerto Ricans residing on the mainland, meanwhile, primarily live in the eastern United States [16]. Puerto Ricans are also considered US citizens, and many have left the island following devastating hurricane damages. Some Central and South Americans have recently left their home countries as political asylees or refugees from violence and economic turmoil; Venezuelans and Guatemalans are among the fastest growing Hispanic subpopulations in the US [16]. 

Evidently, there is wide diversity based on immigration status, language, histories, and cultural assimilation within Hispanic subpopulations. This diversity also extends into both how different Hispanic ethnicities view sexual minorities and the effect of immigration status and culture on the experiences of sexual minorities within these groups. For example, while Mexican Americans, Puerto Ricans, and Cubans share similar attitudes towards sexual minorities on the basis of religion/religious beliefs and culturally-based gender norms, views also differ based on the roles of acculturation and family dynamics between these groups [17, 18]. Furthermore, social factors such as immigration status also exacerbate minority stress experienced by Hispanics who identify as sexual minorities compared to Hispanic sexual minorities with documented status [19]. Thus, intersectional approaches to LGBQ + Hispanic health should be mindful of the vast diversity within the broader Hispanic population. The objectives of this study are to describe and compare differences in health outcomes and access to care within Hispanic subpopulations by LGBQ + status. This study leverages large, nationally representative data from the National Health Interview Survey to explore whether there are sexual orientation-based health disparities within Hispanic sub-ethnicities to help inform targeted and community-based interventions towards achieving LGBQ + Hispanic health equity.

## Data & Methods

### Data & Study Participants

This study used data from the 2013–2018 National Health Interview Survey (NHIS), downloaded from IPUMS at the University of Minnesota [20]. Conducted annually by the National Center for Health Statistics at the Centers for Disease Control and Prevention (CDC), the NHIS is a nationally representative health survey of the civilian, non-institutionalized population that provides timely and comprehensive data on the nation’s health. The family core questionnaire records basic demographic, health, and disability information for each household member while a single random adult in each household is selected for a detailed interview on more specific health information that includes sexual orientation. Beginning in 2013, adult respondents were asked which of the following categories best represents how they identify themselves: lesbian or gay; straight, that is, not gay; bisexual; something else; I don’t know the answer; or they declined to answer the question. We considered sexual minorities as anyone who considered themselves to be lesbian, gay, bisexual, or something else; heterosexuals were defined as anyone who considered themselves as straight. Respondents refusing or not knowing how to answer the question were excluded from the analysis since their sexual orientation identity remains missing.

This study focused on the diversity within the Hispanic sexual minority population. Participants were asked whether they consider themselves to be Hispanic or Latino. If they indicated an affirmative response, participants were asked to provide the number of the following groups that represents their Hispanic/Latino origin or ancestry: 1 Puerto Rican; 2 Cuban or Cuban American; 3 Dominican (Republic); 4 Mexican; 5 Mexican American; 6 Central or South American; 7 Other Latin American; and 8 Other Hispanic/Latino/Spanish. Because of small sample sizes, we combined Dominicans (response number 6) with other Latin American, Hispanic, Latino, and Spanish ancestries (response numbers 7 and 8).

We restricted our analysis to adults aged 18 years and older with sexual orientation and Hispanic ethnicity information provided. Of note, detailed Hispanic identity information is not available in the public use files following a redesign of the NHIS in 2019. Our final analytic sample included Hispanic sexual minority respondents (*n* = 768) who reported their ethnicity as Mexican (*n* = 244), Mexican American (*n* = 172), Puerto Rican (*n* = 123), Cuban (*n* = 42), Central/South American (*n* = 120), and other Hispanic identities (*n* = 67). The comparison group was comprised of respondents indicating their sexual orientation as heterosexual (*n* = 26,036) and reported their ethnicity as Mexican (*n* = 9,562), Mexican American (*n* = 5,994), Puerto Rican (*n* = 2,660), Cuban (*n* = 1,344), Central/South American (*n* = 4,236), and other Hispanic identities (*n* = 2,240).

### Health & Access to Care Outcomes

We assessed an array of self-reported health outcomes and barriers to care. The health outcomes examined in this study included self-rated health as poor or fair (versus excellent, very good, or good health); reporting any of ten chronic health conditions (i.e., hypertension, coronary heart disease, stroke, diabetes, cancer, arthritis, hepatitis, kidney disease, asthma, and chronic obstructive pulmonary disease); and having moderate to severe psychological distress based on Kessler’s six-item evaluation of non-specific psychological distress [21]. Then, we examined the following barriers to care: no usual source of care; unmet medical care due to cost; and unmet mental health care due to cost.

### Statistical Analyses

First, we estimated descriptive statistics to characterize the sample by sexual minority status and Hispanic ethnicity. We used chi-squared tests to compare age category, self-reported sex, relationship status, the presence of children in the household, survey language, citizenship status, educational attainment, and US Census region of residence across Hispanic identities separately for heterosexual and sexual minority adults. Then, we estimated the prevalence for each outcome and used logistic regression models to compare all health and access to care outcomes between heterosexual and sexual minority adults within each Hispanic identity subset. Adjusted logistic regression models controlled for age category, self-reported sex, relationship status, the presence of children in the household, survey language, educational attainment, US Census region, and survey year. Regression results are presented with odds ratios (OR) and 95% confidence intervals (CI). All analyses were conducted using Stata version 18 [22] with survey weights to account for the complex survey design and to report nationally representative estimates. The < BLINDED > University institutional review board deemed this research exempt because all data were de-identified and publicly available through secondary sources.

## Results

Table 1 presents the demographic and socioeconomic characteristics of *heterosexual* adults by self-reported Hispanic ethnicity. Mexican American heterosexual adults tended to be younger than other Hispanics, while Cuban adults were more likely to be 65 years and older. There were no statistically significant differences by sex across heterosexual Hispanic ethnicities. More than half of heterosexual Hispanic adults were married or living with a partner, and heterosexual Mexican adults were least likely to have a child in the household. Heterosexual Cubans (48.9%) were most likely to complete the NHIS survey entirely in Spanish, followed by Mexicans (32.4%) and Central/South Americans (27.6%). Nearly all heterosexual Puerto Ricans (98.8%) were citizens, followed by heterosexual Mexican Americans (92.7%); heterosexual Mexicans (46.8%) were least likely to be citizens. Heterosexual Cubans (26.3%) were more likely to have a college degree, and heterosexual Mexicans (8.5%) were least likely to have a college degree compared to other Hispanic groups. More than 40% of heterosexual Puerto Ricans and adults reporting other Hispanic identities resided in the northeastern US. Over 80% of heterosexual Cubans resided in the southern US, and more than half of heterosexual Mexicans and Mexican Americans resided in the western US.

**Table 1:** Demographic and socioeconomic characteristics of heterosexual adults by Self-Reported Hispanic ethnicity

Sample Size	Mexican	Mexican American	Puerto Rican	Cuban	Central / South American	Other / Multiple Hispanic	*P* Value
	(*n*=9,562)	(*n*=5,994)	(*n*=2,660)	(*n*=1,344)	(*n*=4,236)	(*n*=2,240)	
**Age, years**							
18-25	16.6	27.6	15.9	11.2	17.3	18.3	<0.001
26-34	21.2	21.6	18.5	16.7	21.3	21.4	
35-49	36.1	24.5	27.3	27.3	32.8	25.8	
50-64	18.7	16.2	23.1	23.8	19.8	21.1	
≥65	7.5	10.1	15.2	21.1	8.8	13.4	
Missing data	0	0	0	0	0	0	
**Sex**							
Female	49.7	49.5	51	45.7	50.1	53	0.102
Male	50.3	50.5	49	54.3	49.9	47	
**Relationship Status**							
Married or living with a partner	66.9	54.6	55.1	62.3	61.5	51.2	<0.001
Divorced, separated or widowed	11.7	12.7	18.3	18.1	13.1	18.7	
Never married	21.3	32.7	26.5	19.6	25.4	29.6	
Missing data	0.2	0.1	0.1	0.1	0.1	0.5	
**Children Present in the Household**							
No	38.7	47.7	57.8	63.2	47.6	55.4	<0.001
Yes	61.4	52.3	42.2	36.8	52.4	44.6	
**Survey Language**							
English	50.5	84.3	83.3	39	57.3	75.5	<0.001
Spanish	32.4	5.7	10.2	48.9	27.6	16.4	
English/Spanish or Other Languages	17.1	10	6.5	12	15.1	8.2	
**Citizenship Status**							
Citizen	46.8	92.7	98.8	72.2	56.1	83.2	<0.001
Non-Citizen	51.9	7.1	1.1	27.7	43.3	16.5	
Missing data	1.3	0.2	0.1	0.1	0.6	0.3	
**Educational attainment**							
Less than high school	45.6	20.6	21.3	18.3	26.5	20.8	<0.001
High school graduate	25.3	29.7	28.3	30.6	23.8	26	
Some college	19.2	35.9	32.8	24.6	26.5	31.2	
College graduate	8.5	13.4	17.1	26.3	22.3	21.2	
Missing data	1.4	0.5	0.5	0.2	0.9	0.8	
**Census Region**							
Northeast	4	1.1	46.6	6.4	22.1	41.2	<0.001
Midwest	11.8	9.3	11	3.4	5.6	5.8	
South	31.5	37.3	33.5	84.4	45.6	24.7	
West	52.7	52.3	8.9	5.8	26.7	28.4	

Source: 2013–2018 National Health Interview Survey (NHIS)

Table 2 presents demographic and socioeconomic characteristics for *sexual minority* adults by self-reported Hispanic ethnicity. For each Hispanic subgroup, over half of sexual minorities were younger and aged 18–34 years– except for sexual minority Cubans; fewer than 18% of sexual minority Cubans were younger than 34 years. There were no statistically significant differences in relationship status, but sexual minority Cubans (51.5%) were more likely to report being married or living with a partner than other Hispanic groups. There were also no statistically significant differences in having a child present in the household, and most sexual minority Hispanic adults did not have a child present in the household. Sexual minority Cubans (35.3%) were most likely to complete the NHIS entirely in Spanish, followed by sexual minority Mexicans (19.4%) and Central/South Americans (16.5%). A majority of sexual minority Hispanics were citizens, but sexual minority Mexicans (33.2%) and Central/South Americans (33.9%) were most likely to be non-citizens. Sexual minority Cubans (40.8%) and Central/South Americans (41.8%) were approximately twice as likely to have a college degree compared to all other Hispanic sub-ethnic groups. Most sexual minority Cubans and Mexicans resided in the southern and western US, respectively. A plurality of sexual minority Puerto Ricans resided in the northeastern US, while over 80% of sexual minority Mexican Americans resided in the southern and western US.

**Table 2:** Demographic and socioeconomic characteristics of sexual minority adults by Self-Reported Hispanic ethnicity

Sample Size	Mexican	Mexican American	Puerto Rican	Cuban	Central / South American	Other / Multiple Hispanic	*P* Value
	(*n*=244)	(*n*=172)	(*n*=123)	(*n*=42)	(*n*=120)	(*n*=67)	
**Sexual Orientation**							
Gay/Lesbian	49.3	63.5	55.6	69.9	60.3	57.6	0.29
Bisexual	37	28.1	39.1	20.2	25.2	28.1	
Something else	13.7	8.4	5.3	9.9	14.6	14.4	
**Age**,** years**							
18-25	26.2	36.4	38.5	12.7	31	25.3	<0.001
26-34	28.5	20.3	29.2	4.8	23.4	36.8	
35-49	29.6	23.7	22.3	16	31.2	12.7	
50-64	13.4	15.2	6.9	57.3	12.5	23.4	
≥65	2.3	4.4	3.2	9.2	1.9	1.8	
Missing data	0	0	0	0	0	0	
**Sex**							
Female	51.4	54.1	54.9	34.3	49.6	51.6	0.72
Male	48.6	46	45.1	65.7	50.4	48.4	
**Relationship Status**							
Married or living with a partner	42.9	37.9	31.8	51.5	41.3	42.1	0.49
Divorced, separated or widowed	12.5	10.8	6.7	14.7	10.1	8.3	
Never married	44.6	51.3	61.4	33.9	48.6	47.9	
Missing	0	0	0	0	0	1.7	
**Children Present in the Household**							
No	67.2	75.9	73.2	77.5	79.3	87.1	0.17
Yes	32.8	24.1	26.8	22.5	20.7	12.9	
**Survey Language**							
English	69.9	95.3	94.2	56.4	76.2	89.8	<0.001
Spanish	19.4	2	1.1	35.3	16.5	4.1	
English/Spanish or Other Languages	10.8	2.8	4.7	8.3	7.3	6.1	
**Citizenship Status**							
Citizen	66.8	91.2	99.2	80.6	64.8	89.7	<0.001
Non-Citizen	33.2	7	0.8	19.4	33.9	10.3	
Missing data	0	1.8	0	0	1.3	0	
**Educational attainment**							
Less than high school	29.3	13.5	21.3	6.6	12.4	16.1	0.03
High school graduate	22.6	29	28.9	24	15	18	
Some college	27.8	37.3	28.6	27.9	30.9	43.7	
College graduate	20.1	20.1	21.2	40.8	41.8	22.3	
Missing	0.2	0	0	0.8	0	0	
**Census Region**							
Northeast	4.1	3.4	47.5	12.7	14.3	29.6	<0.001
Midwest	14.2	12.8	12.1	0	5	9.6	
South	26.1	42.2	31.6	83.2	37.8	26.3	
West	55.6	41.6	8.8	4.2	43	34.5	

Source: 2013–2018 National Health Interview Survey (NHIS)

Table 3 presents differences in self-reported health and access to care outcomes by sexual minority status within each Hispanic ethnicity. After controlling for sociodemographic characteristics, sexual minority Mexicans were significantly (*p* < 0.05) more likely to report poor/fair health (OR = 1.88; 95% CI = 1.16–3.05), having at least one chronic health condition (OR = 1.63; 95% CI = 1.07–2.50), exhibiting moderate to severe psychological distress (OR = 3.35; 95% CI = 2.37–4.72), and having unmet health care needs due to cost (OR = 6.46; 95% CI = 2.84–14.71) compared to heterosexual Mexicans. Sexual minority Mexican Americans were significantly more likely to report moderate to severe psychological distress (OR = 2.32; 95% CI = 1.38–3.91), having no usual source of care (OR = 1.78; 95% CI = 1.38–3.91), and unmet mental health care needs due to cost (OR = 2.61; 95% CI = 1.35–5.02) compared to their heterosexual Mexican Americans after controlling for sociodemographic factors. Sexual minority Mexican Americans were also marginally (*p* < 0.10) more likely to report having at least one chronic health condition (OR = 1.84; 95% CI = 0.99–3.43) and unmet medical care needs due to cost (OR = 1.73; 95% CI = 0.93–3.23) than their heterosexual peers.

**Table 3:** Differences in Self-Reported health & access to care by sexual minority status within Hispanic ethnicities

	Mexican	Mexican American	Puerto Rican	Cuban	Central / South American	Other / Multiple Hispanic Identities
**Poor/Fair Health**						
Heterosexual (%)	14.3	13	18.9	14.3	9.9	14.5
Sexual Minority (%)	16.8	7.7	18.3	21.3	8.6	27.6
Unadjusted Odds Ratio	1.21 (0.79-1.84)	0.56 (0.32-0.99)*	0.96 (0.51-1.82)	1.63 (0.75-3.52)	0.85 (0.39-1.82)	2.24 (0.93-5.39)
Adjusted Odds Ratio	1.88 (1.16-3.05)*	0.59 (0.28-1.21)	1.80 (0.94-3.47)†	2.33 (0.82-6.59)	1.18 (0.55-2.54)	4.48 (1.54-13.01)*
**Any Chronic Health Conditions**						
Heterosexual (%)	34	38.3	50.8	44.8	32.2	40.6
Sexual Minority (%)	37.6	46.9	42.3	76.8	28.4	45
Unadjusted Odds Ratio	1.17 (0.78-1.75)	1.42 (0.89-2.28)	0.71 (0.42-1.19)	4.07 (1.78-9.31)*	0.83 (0.51-1.36)	1.20 (0.60-2.40)
Adjusted Odds Ratio	1.63 (1.07-2.50)*	1.84 (0.99-3.43)†	1.46 (0.82-2.60)	4.52 (1.57-13.06)*	1.13 (0.64-2.00)	1.75 (0.88-3.48)
**Moderate to Severe Psychological Distress**						
Heterosexual (%)	19	21.1	26.4	17.2	20	23.9
Sexual Minority (%)	41.8	40.3	37.2	35.1	31.9	32.9
Unadjusted Odds Ratio	3.07 (2.19-4.30)**	2.52 (1.58-4.03)**	1.65 (1.02-2.65)*	2.61 (1.13-6.04)*	1.88 (1.18-2.99)*	1.57 (0.80-3.06)
Adjusted Odds Ratio	3.35 (2.37-4.72)**	2.32 (1.38-3.91)*	1.50 (0.88-2.56)	2.39 (1.00-5.72)†	1.77 (1.08-2.90)*	1.42 (0.70-2.86)
**No Usual Source of Care**						
Heterosexual (%)	32.4	23.7	19.7	23.8	28.5	23.5
Sexual Minority (%)	32.7	36.1	27	14.8	24.4	25.6
Unadjusted Odds Ratio	1.02 (0.70-1.47)	1.82 (1.10-3.00)*	1.51 (0.81-2.81)	0.55 (0.19-1.61)	0.81 (0.46-1.41)	1.12 (0.56-2.26)
Adjusted Odds Ratio	0.98 (0.66-1.44)	1.78 (1.06-2.99)*	1.09 (0.61-1.97)	0.58 (0.20-1.67)	0.81 (0.42-1.55)	0.79 (0.35-1.74)
**Unmet Medical Care Due to Cost**						
Heterosexual (%)	8.2	6.6	6.9	6.8	7.8	7.9
Sexual Minority (%)	10.6	12.3	10.3	15.5	7.5	14.3
Unadjusted Odds Ratio	1.33 (0.88-2.01)	2.00 (1.12-3.58)*	1.54 (0.77-3.06)	2.52 (0.89-7.12)	0.95 (0.41-2.22)	1.94 (0.89-4.24)
Adjusted Odds Ratio	1.37 (0.93-2.03)	1.73 (0.93-3.23)†	1.39 (0.66-2.96)	2.59 (0.76-8.78)	0.91 (0.37-2.22)	1.49 (0.64-3.49)
**Unmet Mental Health Care Due to Cost**						
Heterosexual (%)	1.5	2.3	2.4	1.3	1.8	2.7
Sexual Minority (%)	9.2	7.3	6.4	2.9	8.5	7.7
Unadjusted Odds Ratio	6.72 (3.08-14.64)**	3.29 (1.72-6.29)**	2.82 (1.14-7.00)*	2.27 (0.29-17.76)	5.01 (1.82-13.78)*	2.97 (1.09-8.08)*
Adjusted Odds Ratio	6.46 (2.84-14.71)**	2.61 (1.35-5.02)*	2.72 (0.93-7.99)†	2.22 (0.20-25.24)	4.59 (1.56-13.50)*	1.80 (0.51-6.35)

Source: 2013–2018 National Health Interview Survey (NHIS). **p < 0.001; *p < 0.05; †*p* < 0.10. The prevalence (%) of each outcome was estimated using survey weights and not adjusted for covariates. Odds ratios were obtained from logistic regression models using survey weights. Adjusted odds ratios controlled for age category, sex, relationship status, the presence of children in the household, survey language, educational attainment, US Census region, and survey year

After controlling for sociodemographic characteristics, sexual minority Puerto Ricans were marginally (*p* < 0.10) more likely to have unmet mental health care needs due to cost (OR = 2.72; 95% CI = 0.93–7.99) than their heterosexual peers (Table 3). Sexual minority Cubans were significantly (*p* < 0.05) more likely to have at least one chronic health condition (OR = 4.52; 95% CI = 1.57–13.06) and marginally more likely to have moderate to severe psychological distress (OR = 2.39; 95% CI = 1.00-5.72) than heterosexual Cubans in fully adjusted models. Sexual minority Central/South Americans were significantly more likely to have moderate to severe psychological distress (OR = 1.77; 95% CI = 1.08–2.90) and unmet mental health care needs due to cost (OR = 4.59; 95% CI = 1.56–13.50) than their heterosexual counterparts. Finally, after controlling for sociodemographic characteristics, sexual minority Hispanics of other non-specified ethnicities were significantly more likely to report poor/fair self-rated health (OR = 4.48; 95% CI = 1.54–13.01) compared their heterosexual peers after adjusting for sociodemographic characteristics.

## Discussion

This study compares health outcomes and access to care between sexual minority and heterosexual adults by Hispanic sub-ethnicities. We found similarities and differences in the types of disparities experienced by sexual minority Hispanics– pointing to the need for intersectional research that recognizes Hispanic populations are not a monolith. The diverse histories and sociopolitical experiences of Hispanic subpopulations may lead to variation in discrimination, social connectedness, and documentation status. Moreover, structural and interpersonal discrimination experienced independently by Hispanics and sexual minorities may exacerbate health disparities at the intersections of sexual orientation and Hispanic identity.

There are several possible explanations for why different sexual minority Hispanic subpopulations experienced similar or different health disparities. For instance, we found that sexual minority Mexicans, Mexican Americans, Cubans, and Central/South Americans, were more likely to have higher levels of psychological distress compared to their corresponding heterosexual peers. A prior study found that ethnicity-based discrimination was highest among Hispanics of Mexican, Central American, or Puerto Rican descent– yet lowest among Hispanics of Cuban heritage [23]. This illustrates how the compounding effects of ethnicity and sexuality-based discrimination could create a double disadvantage that leads some Hispanic sexual minorities like those of Mexican and Central American descent, to experience the increased levels of psychological distress found in this study. Differences in findings for Cubans and Puerto Ricans (of note, both share Caribbean descent) may be attributed to the differential experiences between heterosexual and sexual minority populations within those groups, but further research is needed.

Another possible explanation for the significant likelihood of moderate to severe psychological distress across Hispanic sexual minority subgroups could be that citizenship and documentation status may hinder access to care, including mental health care. For instance, recent immigrants must wait five years before they are eligible for Medicare or Medicaid, and undocumented immigrants are ineligible for public health insurance. Of note, the NHIS does not collect information on the number of years immigrants have resided in the United States or documentation status. Some Hispanic subpopulations may be at greater risk of lacking health insurance and a usual source of care. As a result, disparities commonly experienced by Hispanics such as shared decision making, language barriers [24], provider-based cultural competency [25], and health literacy [26] may be made worse in combination with stigma surrounding sexual orientation in medicine [27]. Such communities can be affected by double discrimination in health care experienced by LGBQ + and Hispanics simultaneously [28]. Future studies should continue to leverage community-based research, large-scale quantitative surveys, and qualitative evidence to help inform targeted interventions that advance LGBQ + Hispanic health equity.

Interestingly, across multiple health outcomes surveyed in this study — such as poor/fair health, unmet medical care due to cost, and having no usual source of care — very few sexual minority Hispanic subpopulations reported an increased odds of experiencing each disparity compared to their heterosexual peers. This demonstrates how variation within different sexual minority Hispanic subpopulations may exist and that grouping Hispanic populations together in research may mask such differences. Treating Hispanics as a monolithic group can weaken public health research and proposed solutions. In fact, researchers, policymakers, and practitioners may, unfortunately, develop research-informed solutions that may misrepresent specific subpopulations. Further non-monolithic approaches to research should help identify best practices for achieving health equity at the intersections of sexual orientation and Hispanic identity.

Finally, due to small sample sizes, we were unable to examine further stratifications by sex, which may hide important health disparities experienced by sexual minority Hispanic men and women. For example, previous research has found that sexual minority Hispanic women have a greater likelihood of experiencing less sleep compared to their heterosexual peers while Hispanic sexual minority men have higher rates of HIV testing [29]. Differences can also extend into cardiovascular health and risk-taking behaviors, with another study noting that sexual minority Hispanic women exhibited worse cardiovascular health compared to their heterosexual peers, while no difference was found between sexual minority and heterosexual Hispanic men [30]. Yet, consistent with the large body of research on Hispanic health, these studies did not examine differences within Hispanic subgroups, presenting an additional gap and need for further, non-monolithic approaches.

### Limitations

There were several limitations to this study that provide context to the results and facilitate the interpretation and application of its results. Some key limitations to this study are the potential of self-reporting bias for several personal identifiers (e.g., disclosing accurate sexual orientation or Hispanic ethnicity), relatively small sample sizes which may mask disparities with imprecise results, the years of the data analyzed, lack of assessment of other sexual minorities besides LGB + individuals (e.g., asexual or queer identities), and other limitations associated with NHIS survey methodology (e.g., only one random adult is selected for interview).

While surveys like the NHIS collect rich population-based data, they do so through in-person interviews. This presents a challenge when asking about a person’s identity, such as sexual minority status, Hispanic ethnicity, and citizenship status. Some respondents– especially the most vulnerable participants– may not feel comfortable sharing personal information. This may underreport health issues faced by sexual minorities within Hispanic communities and undocumented immigrants (where stigma may be rampant). A previous study found that bisexual men were more likely to conceal their sexual orientation identity compared to gay men, which attenuated the precision of measuring suicidal ideation disparities among bisexual men [31]. This may present an even greater threat to internal validity when multiple intersecting identities are at play. Specifically, the intersection of cultural norms associated with gender-based roles (e.g., *machismo*) and traditional Hispanic values may make it even more likely that some Hispanic sexual minorities conceal their sexual orientation due to fear of further stigmatization, isolation, or shame. Thus, our health disparity results relying on self-reported survey data may be narrower than possible wider disparities present in reality [32, 33]. Relatedly, self-reporting and recall bias also threaten the reliability of self-rated health outcomes and access to care.

Additionally, the NHIS’s omission of data from certain populations also decreases the external validity of this study. For example, the NHIS does not include data from institutionalized (e.g., incarcerated) or transgender populations. Previous studies have found that, compared to other sexual minorities, transgender individuals have increased barriers accessing health care, higher prevalence of disability, and are at greater risk of being impoverished [34]. The omission of gender identity data collection hinders researchers from understanding the experiences of transgender adults within different Hispanic subpopulations.

Another limitation included relatively small sample sizes for different LGBQ + Hispanic subpopulations. Our analysis was limited to the number of NHIS respondents who self-reported being a sexual minority and Hispanic, which are both minority populations in the United States. For instance, small samples precluded the examination of Dominicans separately from other Latin American, Hispanic, Latino, and Spanish ancestries. The NHIS should consider further oversampling Hispanic subpopulations to strengthen the internal and external validity of a study– especially as the Hispanic populations continues to grow in the United States.

Lastly, this study was limited to data collected between years 2013 and 2018. A major redesign of the NHIS in 2019, and the COVID-19 pandemic in 2020 created disruptions to the NHIS. Moreover, sample sizes were larger in 2013–2018 which facilitated the release of Hispanic ethnicity data in ways that protected participant confidentiality. Despite these limitations, this is among the first studies to examine health disparities and barriers to care among sexual minorities by specific Hispanic ethnicity.

## Conclusion

This study used large-scale and nationally representative data to compare health outcomes and access to care between sexual minority and heterosexual adults by detailed Hispanic ethnicity. We found that sexual minority Hispanic adults– like their heterosexual Hispanic peers — are very diverse with varying levels of educational attainment, geographic location, citizenship status, and age. Results presented here also demonstrate the vast diversity in health disparities within the sexual minority Hispanic community– thus, non-monolithic approaches in Hispanic health *and* sexual minority research should be considered when possible.
